# Left ventricular apex rupture in STEMI

**DOI:** 10.1002/ccr3.4332

**Published:** 2021-07-23

**Authors:** Amr Mohamed

**Affiliations:** ^1^ Department of Internal Medicine Rochester General Hospital Rochester NY USA

**Keywords:** mechanical complications, STEMI

## Abstract

The key clinical message is to know that mechanical complications of myocardial infarction are rare but fatal. A high index of suspicion facilitates diagnosis. The presence of acute heart failure should ring an alarm. Other red flags are cardiogenic shock, new murmur, or evidence of hypoperfusion.

## Graphical abstract

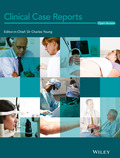

Mechanical complications of myocardial infarction are rare. However, the consequences are catastrophic if missed; here, we present a case highlighting the red flags that can guide the diagnosis of mechanical complications of MI.

A 56‐year‐old man with a past medical history of diabetes presented with chest pain of 23‐hour duration. His BP was 150/80, pulse 120, and SO2 89%. His examination was remarkable for congested neck veins but no murmurs. Chest auscultation showed bubbling crepitation.

EKG revealed anterior STEMI. Because of acute severe heart failure, we performed a bedside echocardiogram. It showed ejection fraction (EF) of 20% and perforated LV apex with apical pseudoaneurysm. There was effusion around the right ventricle (RV) with tamponade. Emergency coronary angiography revealed total proximal left anterior descending (LAD) artery occlusion. Emergency cardiac surgery was performed to reconstruct the LV apex and revascularize the LAD by venous graft. The rest of the hospital course stay was uneventful.

The critical clinical message is to know that mechanical complications are rare. A high index of suspicion facilitates diagnosis. The presence of acute heart failure should ring an alarm. Other red flags are cardiogenic shock, new murmur, or evidence of hypoperfusion (Video [Link], [Link]).

## ETHICAL APPROVAL

Patient verbal consent had been obtained to use the video material.

## CONFLICT OF INTEREST

None declared.

## AUTHOR CONTRIBUTIONS

AM: had contributed to the design and implementation of the research, to the analysis of the results, and to the writing of the manuscript.

## CONSENT STATEMENT

Published with written consent of the patient.

## Supporting information

Video S1

Supplementary Material

## Data Availability

The data that support the findings of this study are openly available in the Journal of the American College of Cardiology at https://doi.org/10.1016/s0735‐1097(00)00879‐2.

