# Supplementary material for: Left ventricular apex rupture in STEMI
Source: Clin Case Rep. 2021 Jul 23;9(7):e04332. doi: 10.1002/ccr3.4332 (PMC8299084; doi:10.1002/ccr3.4332)
Supplement: Supplementary file 2 — Supplementary Material [file CCR3-9-e04332-s002.docx]

**VIDEO S1.** The video shows a transthoracic echocardiogram, apical four-chamber view showing EF of 20%, and perforated LV apex with apical pseudoaneurysm. There is effusion mainly around the right ventricle with tamponade in the form of RV diastolic collapse.
